# From bench to bedside: The mGluR5 system in people with and without Autism Spectrum Disorder and animal model systems

**DOI:** 10.1038/s41398-022-02143-1

**Published:** 2022-09-20

**Authors:** Cornelia Carey, Nisha Singh, Joel T. Dunn, Teresa Sementa, Maria Andreina Mendez, Hester Velthuis, Andreia C. Pereira, Charlotte Marie Pretzsch, Jamie Horder, Stefan Hader, David J. Lythgoe, Diana-Georgina Rotaru, Anthony Gee, Diana Cash, Mattia Veronese, Declan Murphy, Grainne McAlonan

**Affiliations:** 1 Department of Forensic and Neurodevelopmental Sciences, Institute of Psychiatry, Psychology and Neuroscience, King’s College London, De Crespigny Park, London, SE5 8AF UK; 2 Department of Neuroimaging, Institute of Psychiatry, Psychology & Neuroscience, King’s College London, De Crespigny Park, London, SE 5 8AF UK; 3 School of Biomedical Engineering & Imaging Sciences, 4th floor Lambeth Wing, St Thomas’ Hospital, King’s College London, London, SE1 7EH UK; 4 PET Centre, School of Biomedical Engineering and Imaging Sciences, St Thomas’ Hospital, King’s College London, London, SE1 7EH UK; 5 NIHR Maudsley Biomedical Research Centre, Institute of Psychiatry, Psychology & Neuroscience, King’s College London, De Crespigny Park, London, SE 5 8AF UK; 6 Autism Assessment and Behavioural Genetics Clinic, South London and Maudsley NHS Foundation Trust, Bethlem Royal Hospital, Beckenham, UK; 7 Coimbra Institute for Biomedical Imaging and Translational Research (CIBIT), ICNAS, Polo 3 Azinhaga de Santa Comba, 3000-548 Coimbra, Portugal; 8 Centre for Biomarker Research and Imaging for Neuroscience, James Black Centre, Denmark Hill, London, UK; 9 Sackler Institute for Translational Neurodevelopment, Institute of Psychiatry, Psychology and Neuroscience, King’s College London, London, UK

**Keywords:** Molecular neuroscience, Autism spectrum disorders

## Abstract

The metabotropic glutamate receptor 5 (mGluR5) is a key regulator of excitatory (E) glutamate and inhibitory (I) γ-amino butyric acid (GABA) signalling in the brain. Despite the close functional ties between mGluR5 and E/I signalling, no-one has directly examined the relationship between mGluR5 and glutamate or GABA in vivo in the human brain of autistic individuals. We measured [^18^F] FPEB (^18^F-3-fluoro-5-[(pyridin-3-yl)ethynyl]benzonitrile) binding in 15 adults (6 with Autism Spectrum Disorder) using two regions of interest, the left dorsomedial prefrontal cortex and a region primarily composed of left striatum and thalamus. These two regions were mapped out using MEGA-PRESS voxels and then superimposed on reconstructed PET images. This allowed for direct comparison between mGluR5, GABA + and Glx. To better understand the molecular underpinnings of our results we used an autoradiography study of mGluR5 in three mouse models associated with ASD: *Cntnap2* knockout, *Shank3* knockout, and *16p11.2* deletion. Autistic individuals had significantly higher [^18^F] FPEB binding (*t* (13) = −2.86, *p* = 0.047) in the left striatum/thalamus region of interest as compared to controls. Within this region, there was a strong negative correlation between GABA + and mGluR5 density across the entire cohort (Pearson’s correlation: *r* (14) = −0.763, *p* = 0.002). *Cntnap2* KO mice had significantly higher mGlu5 receptor binding in the striatum (caudate-putamen) as compared to wild-type (WT) mice (*n* = 15, *p* = 0.03). There were no differences in mGluR5 binding for mice with the *Shank3* knockout or *16p11.2* deletion. Given that *Cntnap2* is associated with a specific striatal deficit of parvalbumin positive GABA interneurons and ‘autistic’ features, our findings suggest that an increase in mGluR5 in ASD may relate to GABAergic interneuron abnormalities.

## Introduction

The metabotropic glutamate receptor 5 (mGluR5) is an important regulator of excitatory (glutamate) and inhibitory (γ-aminobutyric acid, GABA) pathways [1, 2] and atypicalities in its expression are associated with a number of neurological and psychiatric conditions including epilepsy [3], anxiety [4] and autism spectrum disorder (ASD) [5]. Human brain mGluR5 has been implicated in early neurodevelopmental processes and is present from the ninth gestational week [6]. The mGlu5 group 1 receptors are mainly found on postsynaptic terminals of neurons and on glial cells [7] and are functionally and physically linked to inotropic N-methyl-D-aspartate (NMDA) receptors [8–10]. While mGluR5 activity primarily enhances glutamate-mediated postsynaptic excitation [11], there is also evidence for this receptor acting presynaptically to enhance neurotransmitter release through Ca^2+^ release and presynaptic Ca^2+^ signalling [12, 13]. Furthermore, glutamate spill over may activate presynaptic mGluR5 resulting in repetitive behaviours [14].

mGluR5 is also closely linked to GABA [15]: in animal studies activation of mGluR5 has been found to both increase [16] and inhibit GABAergic transmission [17], mGluR5 interacts with GABA-A receptors and is co-localized with GABA-A *α*1 subunit-containing receptors in the amygdala, hippocampus and globus pallidus [18–20]. Additionally, GABA has been shown to act as a negative modulator of the mGluR pathway on pharmacological testing of GABA agonists [21]. Thus, mGlu5 receptors may modulate brain development and function through a complex bi-directional interaction with major excitatory (glutamate) and inhibitory (GABA) neurotransmitter systems. No-one has directly examined the relationship between mGluR5 and GABA in vivo in the human brain and no-one has directly examined the relationship between mGluR5 and glutamate in vivo in autistic individuals.

Disruption to these excitatory-inhibitory neural pathways has been strongly implicated in the impaired information processing and social behaviours observed in ASD [22]. ^1^H-MRS studies of ASD have reported differences in glutamate levels [23, 24] and GABA responsivity [25, 26] in those with ASD. Higher cerebellar mGluR5 availability has been reported in ASD in post-mortem [27] and animal studies [28]. There have only been a few small studies of in vivo positron emission tomography (PET) in ASD, one of which included six autistic participants and three controls [5], and identified higher [^18^F] FPEB binding potential in the cerebellum, postcentral gyrus, entorhinal area and precuneus in adults with idiopathic ASD [5]. The others included individuals with Fragile X syndrome [29, 30] which is the most common genetic cause of ASD [31, 32]. For example, Brašić and colleagues reported lower expression of mGluR5 in cortical and subcortical regions in Fragile X syndrome and higher cortical mGluR5 in idiopathic ASD, albeit confounded by differences in IQ between the groups [30]. Therefore, in this study we used PET to examine mGluR5 availability with the radiotracer [^18^F] FPEB (3-[18 F] fluoro-5-[(pyridin-3-yl)ethynyl]benzonitrile in a small sample of adult men with and without idiopathic ASD using two pre-defined regions of interest (ROIs): the left dorsomedial prefrontal cortex and left striatum/thalamus. This allowed for direct comparison between mGluR5 and GABA + (GABA + macromolecules) and Glx (glutamate + glutamine) as the two regions were mapped out using each participant’s MEGA-PRESS voxel on MRI scanning and then applying this precise voxel region to each participant’s reconstructed PET scan, rather than the traditional CIC (Centre for Integrative Connectomics) brain atlas. ROIs were selected based on evidence linking social and cognitive difficulties in ASD to prefrontal and subcortical regions [33–36]. However, our focus was on the striatum/thalamus because we previously showed that this is among the first brain regions to show structural abnormalities in infants who go on to develop ASD [37] and excitation-inhibition balance is disrupted in this region in adults with ASD [38]. We expected to see differences in mGluR5 binding in the whole brain, dorsomedial prefrontal cortex, and left striatum/thalamus in ASD compared to typically developing controls and that mGluR5 levels in these regions would be related to excitatory-inhibitory (glutamate and GABA) metabolite levels.

To improve our understanding of the neurobiological basis of any findings from the human study, we included an autoradiography investigation of mGluR5 binding in three distinct mouse models commonly used in the study of ASD and that have been used previously in our translational studies [26]: *Cntnap2* knockout (KO), *Shank3* KO and *16p11.2 deletion*. The *Cntnap2* gene encodes a large multidomain neuronal adhesion molecule listed as a “strong candidate (2 S)” gene for ASD in the Simons Foundation Autism Research Initiative database (SFARI) (https://gene.sfari.org/)*. Cntnap2* mediates interactions between neurons and glia during nervous system development (https://www.ncbi.nlm.nih.gov/gene/26047) and its expression in mice parallels that observed in humans in the striatum, thalamus, and amygdala [39]. Therefore, animal models of *Cntnap2* are especially useful in exploring the role of mGluR5 in these brain regions. *Shank3* mutations display high frequency and penetrance in individuals with ASD and intellectual disability (1 in 50) [40]. *Shank3* encodes a scaffolding protein located at glutamatergic synapses [41, 42] and intracellular *Shank* proteins are involved in second messenger communication between mGluR5 and NMDA receptors [43]. Abnormalities in subcortical structure and function have been described in *Shank3* KO mice [44] with increased mGluR5 availability reported in the striatum [42] and thalamus [28]. Thus, the *Shank3* KO are expected to replicate mGluR5 findings in the same region in people with ASD. Finally, human chromosome 16p11.2 microdeletion is the most common gene copy number variation in autism accounting for 0.5–1% of all ASD cases [45]. The 16p11.2 region impacts cell migration and synaptic functions [46] and is associated with socio-cognitive impairments in humans and animal models [47]. We conducted the preclinical investigation using mouse models as an exploratory study. We hypothesized that there would be differences in mGluR5 availability using autoradiography between wild type mice and at least one of these three relevant mice models.

## Materials and methods

### Study design

We carried out ^1^H-MRS and PET studies at the Centre for Neuroimaging Sciences, King’s College London and the PET imaging centre, St Thomas’ Hospital London, respectively. We used the MEGA-PRESS sequence [48] to specifically examine GABA + (GABA + macromolecules) and Glx (glutamate + glutamine) complex concentration in male adults with ASD as compared to non-ASD male adults. The radioligand [^18^F] FPEB was used in the PET study to measure mGluR5 availability. Ten autistic participants and eighteen healthy non-ASD participants underwent MRI (magnetic resonance imaging) scans including MRS. Due to logistical issues, out of these participants, six male ASD participants and nine male control participants proceeded to PET scanning. We have reported the data for those who underwent both MRS and PET scanning only. We did not include female participants as we wished to create as homogenous a group as possible. There is some evidence that GABA levels fluctuate with the menstrual cycle [49, 50] and another consideration was a safety concern regarding the risk of exposing reproductive age females to (albeit low dose) radioactivity.

We also performed a quantitative autoradiography study using the radioligand [^3^H] MPEP to estimate mGluR5 density in mouse brain sections from three mutant strains commonly used as ASD models, namely *Cntnap2* KO, *Shank3* KO, and the *16p11.2 deletion* strain.

### Human ^1^H-MRS and PET: Participants and recruitment

The ASD participants were recruited from a research database of King’s College, London (KCL) and the Behavioural Genetics Clinic at the Maudsley Hospital, a national referral service for the diagnosis of neurodevelopmental disorders in adults. Non-ASD participants were recruited via local advertisements.

All participants met the following criteria: 1.) Male and aged between 18**–**60 years old. 2.) Right-handed. 3.) Intelligence Quotient (IQ) score above 70. 4.) Capable of giving written informed consent. 5.) Able to read, comprehend and record information written in English. 6.) Bodyweight < 120 kg and BMI within the range 18.5–33 kg/m^2^ (inclusive). In addition, ASD participants all met ICD-10 criteria for autistic disorder (F84.0) or Asperger’s syndrome (F84.5) and had received a clinical diagnosis of ASD [51] by an experienced psychiatrist. Diagnoses were supported by the Autism Diagnostic Observation Schedule version 2 (ADOS-2) [52] in all participants and where possible (if an informant was available) the Autism Diagnostic Interview – Revised (ADI-R) [53].

IQ was assessed using the English version 2 of the Wechsler Abbreviated Scale of Intelligence (WASI) with four subscales: Vocabulary, Similarities, Block Design and Matrix Reasoning [54].

### ^1^H-MRS data acquisition and processing

Data was obtained at the Centre for Neuroscience, KCL, on a GE 3 T MR 750 System (GE Medical Systems, Chicago, WI, USA) using a 32-channel head coil (Nova Medical Systems, Wilmington, MA, USA). Total scan time was 60 minutes including all protocols. One sagittal T1-weighted high resolution ADNI Go Inversion Recovery Spoiled Gradient Recalled (IR-SPGR) anatomical scan was acquired for each participant with repetition time (TR) = 7.312 ms, echo time (TE) = 3.016 ms, inversion time (TI) = 400 ms, flip angle (FA) 11°, field of view 270 mm, 256 × 256 matrix, 200 slices, voxel dimensions (X, Y, Z): 1.055 × 1.055 × 1.2 mm. This anatomical scan was then used to position the ^1^H-MRS voxel and to obtain voxel tissue information for further metabolite quantification (see below). Following the structural scan a single-voxel ^1^H-MRS data was acquired using the MEGA-PRESS [55] sequence with the following parameters: TR = 2000 ms, TE = 68 ms, bandwidth = 5 kHz; number of data points = 4096; sinc-Gaussian modulated editing pulses, duration = 16 ms, editing frequency = 1.9 ppm, control frequency = 7.5 ppm, 352 averages (176 ON and 176 OFF); phase cycle length of two; CHESS water suppression, dimensions: right-left 35 mm; anterior-posterior 30 mm; superior-inferior 25 mm (approximately 27 cm^3^). Additionally, 16 unsuppressed water scans with the same parameters were also acquired for further water-scaling metabolite quantification and eddy-current correction (see below). The voxel was positioned to include the left striatum and thalamus as previously described [38, 56].

Original raw GE P-files were exported from the scanner and pre-processed using in-house scripts adapted from FID Appliance (FID-A) pre-processing pipeline [57]. FID-A runs several steps including weighted receiver coil combination, removal of motion corrupted averages, frequency and phase drift correction, spectral registration to align ON and OFF sub-spectra and obtain the difference spectrum for further analysis [58].

Spectra were then analysed using LCModel 6.3 – 1 L (Stephen Provencher Inc., Oakville, Canada) [59]. LCModel uses a priori knowledge of expected individual metabolite peaks, i.e., a basis set, and fits the model to the experimentally acquired spectra. The basis set for the current work were simulated with FID-A software and high spatial resolution density-matrix simulations [60]. The basis set included GABA, glutamate, glutamine, n-acetylaspartate, n-acetylaspartylglutamate and glutathione. The water unsuppressed signal was used as internal reference, i.e., water-scaling, and to perform eddy current correction. To control for partial volume effects and for the different amounts of ‘visible’ water in each tissue type grey matter (pGM), white matter (pWM) and cerebrospinal fluid (pCSF), which influences the quantification of the metabolites due to the water-scaling procedure, each voxel was segmented to obtain the proportion of each tissue type. This was done using the Gannet 3.0 toolkit (http://www.gabamrs.com/downloads) segmentation routine with SPM8 (https://www.fil.ion.ucl.ac.uk/spm/software/spm8/, University College London, UK) running in MATLAB 9.2.0 (R2017a, The Mathworks Inc., Natick, Massachusetts, USA). These values were then used to correct the individual metabolite levels following the equation:

$$Met_{corr} = {Met_{LCM}} ^\ast \frac{{\left( {43300^\ast pGM + 35880^\ast pWM + 55556^\ast pCSF} \right)/35880}}{{1 - pCSF}}$$

where Met_corr_ is the corrected metabolite value, Met_LCM_ is the original quantification obtained from LCModel, 43300, 35880, and 55556 are the concentrations (in mM) of water in GM, WM, and CSF, respectively [61]; pGM, pWM and pCSF are the voxel proportion of GM, WM, and CSF, respectively. The division by 35880 in the numerator corrects for the initial LCModel analysis that assumes a purely white matter voxel during quantification [62]. No metabolite T_1_ or T_2_ relaxation times corrections were performed, thus concentrations are presented in institutional units (i.u.).

**Fig. 1:**
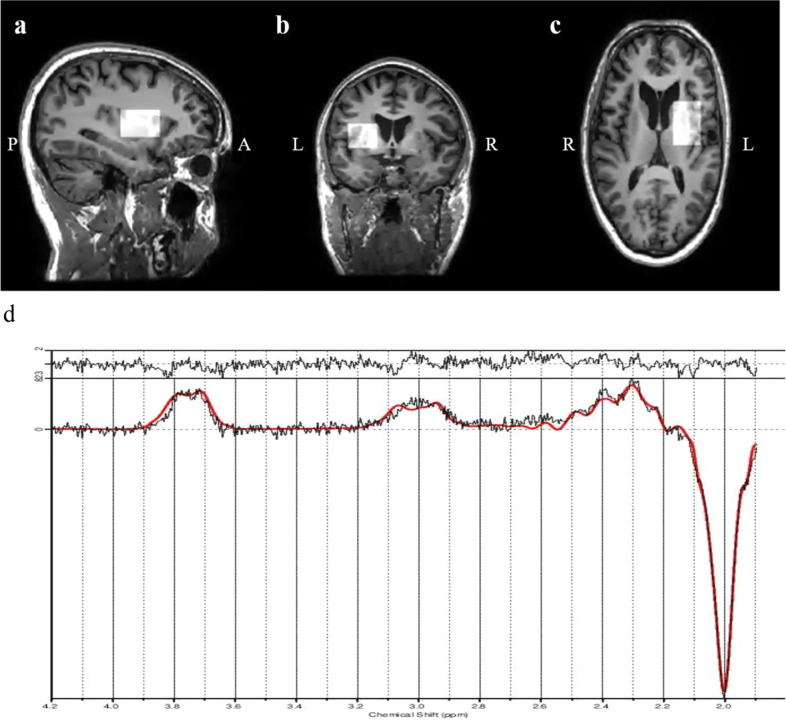
Example of the position of the MEGA-PRESS voxel (3 × 3 × 3 cm^3^) in the left striatum/thalamus. **a** Sagittal view; **b** Coronal view; **c** Axial view; **d** Difference spectrum from a representative participant (black line) and LCModel fit (red line). Glx glutamate+glutamine, GABA + gamma-aminobutyricacid+macromolecules, tNAA n-acetylaspartate+n-acetylaspartylglutamate, MM macromolecules, ppm parts per million.

The measure of glutamate was Glx (glutamine + glutamate) as glutamate alone cannot be reliably detected at 3 T [25]; the GABA measure included macromolecules and is thus referred to as GABA + . Difference spectra were visually inspected for fitting quality. Standard deviation of the Cramer-Rao Lower Bound (%CRLB) for GABA + and Glx measurement was set at a threshold of 20%. SNR (Signal to noise ratio) and linewidth also influence the viability of metabolite quantification [63], and only data with a SNR equal to or above 16 (maximum was 27) were accepted. After thorough data quality assessment, only one dataset was excluded from the final analysis (the SNR was too low, CRLB was too high for both GABA + and Glx and the spectrum was visibly distorted on inspection). The included data had %CRLB ranging from 4 to 6 for GABA + , from 2 to 5 for Glx and SNR ranging from 16 to 27. See Fig. 1.

### PET data acquisition and processing

There was a variable time interval across participants between MRI and PET scanning with seven participants having the MRI and PET scan within 9 days of each other and the other eight participants having both scans within 1-5 months of each other (Average time interval for Controls 21 days vs average ASD 57 days).

The following is a summary of the methods used in the human PET study. For more details, please see the supplementary material.

The radioligand [^18^F] FPEB was synthesised as previously described [64] and a dose of 200 MBq was injected in the participants’ dominant antecubital vein as a 10 s bolus. A low-dose computed tomography was acquired immediately prior to the PET scan for attenuation correction and PET emission data was collected in 3D-mode for a duration of 90 minutes. Images were reconstructed using the VPFX method (fully 3D time-of-flight iterative reconstruction) with frame durations: 1 × 10 s, 10 × 5 s, 6 × 10 s, 3 × 20 s, 87 × 60 s.

Continuous and manual arterial blood samples were also acquired during the scan for deriving whole blood and plasma-parent concentrations for input functions to the kinetic modelling. Continuous arterial blood samples were acquired for the first 15 min following PET scan start with the arterial line (PTFE coated, inner diameter 1 mm) connected to an automated blood sampling system (Allogg ABSS, www.allogg.se, Sweden) and a peristaltic pump set at 5 mL/min. Manual samples were acquired from a three-way tap close to the Allogg detector. Seven 6 mL samples were drawn at 3, 8, 12, 20, 30, 60- and 90 minutes post scan start. These samples were used to measure whole blood tracer concentrations and centrifuged for plasma concentrations to be measured and for metabolite-parent separation. Five additional 0.5 mL manual samples were acquired at 5, 10, 14, 45 and 75 min to improve Allogg-PET cross-calibration and in extrapolating the whole-blood continuous curve to 90 minutes. Plasma parent fractions (ratio of FPEB to all 18 F labelled activity) were fitted to the sigmoid function described by Owen et al. (2014) [65]. The Plasma Parent concentration was calculated by multiplying this fitted parent fraction model with the plasma concentration. This data was used as the plasma-parent input function.

Rather than reporting using a CIC brain atlas, we used three a priori regions: whole brain and MEGAPRESS-defined left striatum/thalamus & dorsomedial prefrontal cortex. Grey matter only was included from those regions to extract [^18^F] FPEB binding availability measures from the PET data within the same region. Time activity curves (TACs) were extracted and fitted by multilinear regression models used previously [66] to estimate Volume of distribution (VT) and Distribution Volume Ratio (DVR), since binding potential (BPnd) was not directly measurable. We focussed on DVR in multilinear reference tissue models (MRTM1) using the cerebellum as a reference region.

### Rodent autoradiography: Overview and animal models

We performed quantitative autoradiography to estimate mGluR5 availability in brain slices from *Cntnap2* KO male mice (Cntanp2 −/−), *Shank3* KO mice (*Shank3* −/−) (Hoffman-La Roche Ltd), and 16p11.2 deletion mice (stock no. 013128, The Jackson Laboratory, USA). Control mice included male WT littermates. In the *Cntnap2* and *16p11.2 deletion* models the sample included *n* = 8 /group, in the *Shank3* model the sample was *n* = 7/group. The sample size was selected based on previous in-house pilot experiments and previously published autoradiography in these mice [26]. No randomisation or blinding was indicated.

To determine mGluR5 availability we used titrated 2-methyl-6-(phenylethynyl)-pyridine [^3^H] MPEP as the radioligand. While the use of [^18^F]-FPEB in both humans and mice would have been preferable, this was not possible for logistical reasons. We have elaborated on the use of [^3^H] MPEP in the discussion below. Mice were euthanised by decapitation, their brains rapidly removed and frozen in cooled isopentane. To approximate the patient characteristics in the human ASD PET study as closely as possible, we used adult (12 weeks of age) male mice in all models [67].

### Rodent autoradiography: protocol

Frozen mouse brains were placed in a cryostat (#Slee, MNT) cooled to −17 ± 2 °C and cut into 15 µm coronal sections at an anatomical level of Bregma 0.98 mm for the striatum (caudate putamen) [68]. The sections were mounted on positively charged slides (VWR#631-0446) and kept frozen at −80 °C until the day of the experiment. On the day of the experiment the slides were warmed to room temperature before the start of the experiment. Sections were incubated for 20 min in 50 mM Tris buffer made up in 0.9% *w/v* NaCl (pH 7.4) at room temperature. They were then incubated with 2 nM of the [^3^H] MPEP (#VT237, ViTrax, USA) in Tris buffer at 4 °C for 60 min to determine total binding. Non-specific binding (NSB) was determined in adjacent sections following the same protocol but with the addition of 10 µM MPEP to [^3^H] MPEP incubation. Following this, the slides were washed twice (2 × 2 min) with buffer at 4 °C, followed by a dip in distilled water, and then left to dry overnight. Once dry, they were exposed to ^3^H-sensitive Amersham Hyperfilm (VWR# 28-9068-50) along with ^3^H microscales (American Radiolabelled Chemicals, #ART0124B) for four weeks before development.

For quantification, ROIs were drawn in the striatum and frontal regions, measured in triplicate, and averaged as is standard practice. Optical density was determined using ImageJ v1.49 (NIH, USA) software. For each mouse, total and non-specific binding were obtained for each mouse and averaged after conversion to nCi/mg using Graphpad Prism v8 (GraphPad software LLC, USA).

### Statistics

Demographic, neuropsychological, voxel tissue composition and spectroscopy data were analysed with IBM SPSS statistics version 23 (IBM Corporation, IL, USA). All data were checked for normal distribution with the Shapiro–Wilk test and for homogeneity of variance with Levene’s test. Between group differences were then investigated with two-tailed independent samples t-test. Correlation analyses between metabolites and DVR were assessed with Pearson’s correlation analysis. For all analyses, α was set at 0.05. Results between groups are reported as uncorrected p-values and would not have survived multiple correction analysis. For the rodent autoradiography, non-specific binding was subtracted from the total binding to give the specific binding values, which were compared between WT and mutant mice using two-way ANOVA corrected for multiple comparisons using Sidak correction.

### Ethics

The [^18^F] FPEB study was approved by West London subcommittee of the National Research Ethics Committee for the UK reference 14/L0/0309.

## Results

### Human study

Nine controls and six autistic participants were included. The two groups did not differ significantly for age and IQ. As expected, there were significantly higher obsessive-compulsive behaviours as rated on the Obsessive-Compulsive Inventory – Revised (OCI-R) in the ASD group compared to the non-ASD group. The difference in OCI-R score did not correlate with mGluR5 DVR or GABA + concentration in the left striatum/thalamus. Please see Table 1 in the supplementary material for further detail regarding demographics and participant characteristics.

### ^1^H-MRS study

**Fig. 2:**
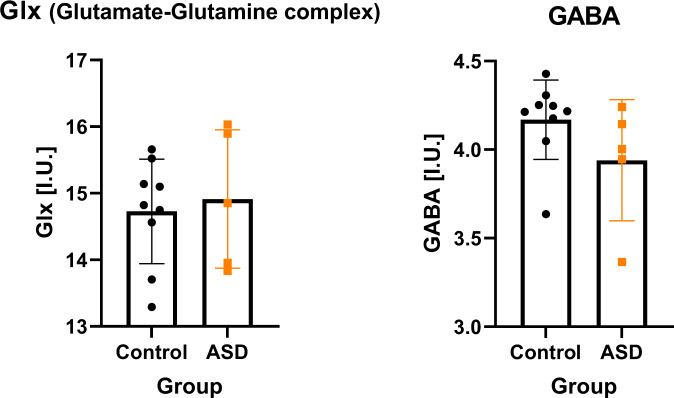
Comparison of striatal/thalamic Glx (Glutamate and Glutamine) and GABA+ complex (GABA + macromolecules) in the ASD and control groups. The control group is represented in black and the ASD (Autism Spectrum Disorder) group is represented in orange. Error bars represent one standard deviation.

For clarity and to remain concise, these results will refer to the left striatum/thalamus only. DMPFC data is included in the supplementary material. There was no difference in voxel tissue composition for grey matter (*t* (13) = 1.97, *p* = 0.07), white matter (*t* (13) = 0.50, *p* = 0.41), or CSF (*t* (13) = 0.32, *p* = 0.44) between the two groups. There were no significant differences between groups for %CRLB for GABA (*t*(12) = −1.39, *p* = 0.37), %CRLB for Glx (*t*(12) = 1.49, *p* = 0.16), SNR (*t*(12) = −0.22, *p* = 0.83), or FWHM (*t*(12) = 0.06, *p* = 0.95). There was no evidence of group differences in estimated Glx levels in the left striatum and thalamus (*t* (12) = −0.38, *p* = 0.71, *d* = −0.19). GABA + levels were lower in the ASD group as compared to non-ASD, but this did not reach statistical significance (*t* (12) = 1.5, *p* = 0.15, *d* = 0.23). See Fig. 2.

### [^18^F] FPEB: Higher mGluR5 availability in ASD group

There were no significant differences in [^18^F] FPEB tracer dosing between the two groups: 167.5 + /− 6.1 MBq in the control group versus 171.8 + /− 13.8 MBq in the ASD group, *p* = 0.4.

Initially we used blood data to calculate V_T_ and DVR based on the MA1 method. Using this method, we were forced to omit three subjects (two autistic participants, one control) as two were missing blood data and one could no longer tolerate the scan after 76 minutes. There was no difference in V_T_ or DVR between groups looking at the whole brain or at the specific MRS-delineated regions of interest (left striatum/thalamus, and dorsomedial prefrontal cortex; all *p* > 0.05).

**Fig. 3:**
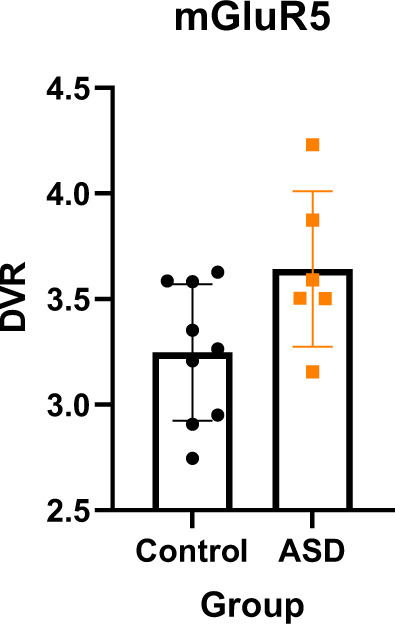
Comparison of striatal/thalamic mGluR5 availability as measured by DVR (Distribution Volume Ratio) in the ASD and control groups. The control group is represented in black and the ASD (Autism Spectrum Disorder) group is represented in orange. Error bars represent one standard deviation.

DVR can also be estimated using a reference region model (MRTM1), here using the cerebellum as the reference region. This increased the power of the study as it allowed us to include the two participants with missing blood data. Additionally, cropping the dynamic PET data to 76 minutes allowed the subject that withdrew at 76 minutes to be included. This allowed us to include the full sample size of nine controls and six autistic participants. Our analysis showed that the ASD group had higher mGluR5 availability as measured using DVR in specific [^18^F] FPEB binding in the whole brain (*t* (13) = 2.46, *p* = 0.029) and using the MRI delineated left striatal/thalamic mask (*t* (13) = −2.86, *p* = 0.047*)*. See Fig. 3.

**Fig. 4:**
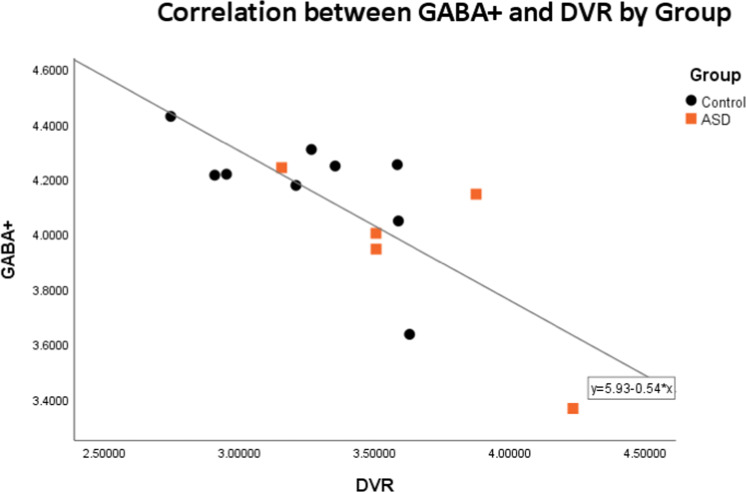
Correlation between GABA + levels and DVR (Distribution Volume Ratio) of mGluR5 in the left striatum and thalamus. The control group is represented in black and the ASD (Autism Spectrum Disorder) group is represented in orange.

There was a strong negative correlation between GABA + levels and mGluR5 availability in the left striatum/thalamus ROI (See Fig. 4) across the whole sample (Pearson correlation: *r*(14)= −0.763, *p* = 0.002) with a trend to significance in the ASD population (Pearson correlation *r*(5) = −0.797, *p* = 0.107).

### Post hoc analysis

One of the autistic participants reported occasional use of Zopiclone for insomnia. Zopiclone is a non-benzodiazepine but binds with high affinity to benzodiazepine receptors [69]. This participant did not present as an outlier for measures of GABA + , Glx, or mGluR5. When this participant was excluded from the analysis the difference between groups for mGluR5 in the left striatum just lost significance (*t*(12) = −2.15, *p* = 0.052) and the strong negative correlation between GABA + and mGluR5 across the whole cohort remained (*r* (13) = −0.761, *p* = 0.003).

### Quantitative autoradiography findings

**Fig. 5:**
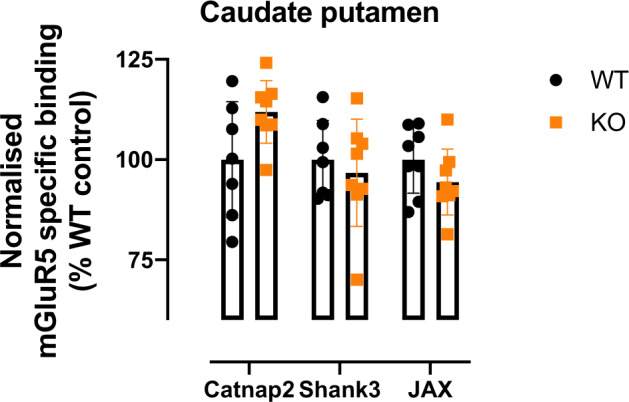
Autoradiography of mGluR5 specific binding in the caudate and putamen of three mouse models associated with ASD (*Cntnap2* knockout, *Shank3* knockout and 16p11.2 deletion (JAX)). Wildtype littermates (WT) are represented in black and knockout (KO) mice are represented in orange. *n* = 7–8/group.

Two-way ANOVA of striatum (Caudate putamen) in all three models showed a trend for interaction between strain and genetic modification (*p* = 0.054). The *Cntnap2* strain showed higher tracer binding (*n* = 15, *p* = 0.03) following multiple corrections. See Fig. 5.

## Discussion

We conducted a translational molecular imaging study of mGluR5 in adults with and without ASD, and in ASD-related rodent models. Our objective was to test if ASD is associated with altered availability of mGluR5 in the whole brain, dorsomedial prefrontal cortex, and striatum/thalamus in vivo; and whether mGluR5 availability is related to the levels of excitatory-inhibitory (glutamate and/or GABA + ) metabolites in the same regions. Similar to previous PET studies investigating mGluR5 in ASD and related conditions [5, 30], we found higher availability in the ASD group albeit in different regions given the differences in methodology. Our results show that adults with idiopathic ASD, but without intellectual disability, have higher availability of striatal/thalamic mGlu5 receptors; the striatum is known to be a region with high mGluR5 density [1, 6] and has been shown to be implicated in both social cognition [1] and ASD [33, 34, 37, 38]. This finding of greater mGluR5 availability was replicated in our study using *Cntnap2* KO mice, a model associated with ASD.

A major strength of this study was the use of the same MEGA PRESS voxels to define the regions of interest in each individual participant’s MRI and PET images, rather than comparing MRS findings to regions defined by a CIC brain atlas. This allowed for a more accurate comparison of mGluR5 and regional brain metabolites. Using this technique, we identified a negative correlation between mGluR5 availability (measured using PET imaging) and GABA + (measured using ^1^H-MRS) in the region of interest containing left striatum and thalamus.

We found a trend towards lower levels of GABA + in our ROI which, like previous adult studies [38], was not statistically significant. The heterogeneity in GABA + levels in our study might explain why GABAergic medications such as low dose benzodiazepines may improve social deficits in some people with ASD but in others cause a paradoxical reaction [70, 71] and still other GABAergic medications such as cyclopyrrolones may worsen core ASD features [72]. The relationship between GABA + and mGluR5 may explain why results from pharmacological studies of drugs targeting mGluR5 in ASD or related conditions mirror the contradictions of GABA drug studies [73–76]. Pharmacological studies in animal models relevant to ASD reflect this complexity and show that autistic-like phenotypes can be rescued when mGluR5 levels are corrected in either direction to achieve a proposed ‘acceptable’ range [74, 75, 77, 78]. Similarly, the use of mGluR5 antagonists in rodents results in varying levels of improvement [79–85] and an increase in social deficits with mGluR5 antagonist treatment in wild-type rats was previously reported [73]. The importance of the relationship between GABA + and mGluR5 is suggested by animal studies which indicate that stimulation of GABAergic systems may alter mGluR5 activity [86–91].

Given the uncertainty regarding the role of mGluR5 in ASD, animal studies can provide invaluable information regarding potential underlying mechanisms of action. Animal studies confirm a crucial role for mGluR5 in early striatal development and the maturation of GABAergic inhibitory systems in particular: blocking mGluR5 during embryonic or postnatal neurogenesis decreases GABAergic cell proliferation and disrupts inhibitory circuits [92, 93]. The mouse studies we conducted in parallel with our human study indicate that *Cntnap2* KO mice, a model used in the study of ASD^1^ [94, 95], have a similar higher mGluR5 availability in the striatum as we observed in the human ASD group. *Cntnap2* deficiency leads to a decrease in dendritic arbours and spines, resulting in an overall change in synaptic network activity due to an imbalance in excitatory/inhibitory connections [96] and is associated with reduced numbers of GABAergic interneurons in the cortex, striatum and hippocampus [95]. *Cntnap2* KO mice have a specific striatal deficit of parvalbumin-positive GABA interneurons and ‘autistic’ features [97]. This raises the possibility that we are seeing higher mGluR5 availability in ASD in response to GABAergic interneuron abnormalities in the ASD group. However, a similar finding was not identified using the *Shank3* KO mice - despite previous reports of increased mGluR5 in the striatum and thalamus [28, 42]. We also found no difference with the 16p11.2 deletion which is implicated in ASD and cortico-striatal functioning [47], although the relationship between 16p11.2 deletion and mGluR5 is less direct and likely to exaggerate the postsynaptic protein synthesis downstream of mGluR5 [98]. As our main aim related to mGluR5, we did not perform a MEGA PRESS analysis in our three rodent models, although this would potentially have made for an interesting comparison with our human study.

There are some limitations to our study. Our human study included small sample size and so the primary analysis had low power to detect a small effect. We did not recruit females; therefore, our findings are limited to adult men. However, this also strengthens our findings given that differences in GABA and mGluR5 have been identified between the sexes[49, 99, 100]. Our ASD population only included individuals with higher-than-average IQ who were free of medical co-morbidities (e.g., epilepsy, where there is considerable evidence of GABAergic dysfunction [101–103]). However, the majority of autistic individuals have normal range IQ [104] and do not have epilepsy [105]. In addition, most spectroscopy studies in adolescents and adults with ASD [23, 38, 56, 106] only include individuals with average or above average IQ and so in this respect our approach is in line with the literature. Nevertheless, while the elimination of medical comorbidities in our sample represents a strength of the study in terms of avoidance of pathophysiological confounds, it also cautions against extrapolation of our findings to the heterogeneous ASD population – e.g., those with intellectual disability. Regarding the data acquisition techniques used, PET and autoradiography can quantify the availability of mGluR5 but not their functionality, for such purpose other methods (e.g., electrophysiology) would be required. There was a variable time interval between MRI and PET studies with a longer average interval among the ASD group. While this was not desirable, our comparison between GABA and mGluR5 was presented across the full group. While we cannot rule out the possibility that the difference in time interval between the groups may have contributed to the trend-only relationship between GABA and mGluR5 in the ASD group, GABA has been shown to demonstrate long-term reproducibility up to seven months [107] so this is unlikely to explain our results.

The differences found in left striatum/thalamus and whole brain DVR should be treated cautiously as biases can arise from shorter dynamic studies and using this in a reference region input model (MRTM1) may also create biases, although it is worth noting that the use of this model allowed us to maximise data collection in a vulnerable population. Multiple comparison corrections have not been made, however only whole brain, left striatum and DMPFC were studied rather than the full CIC atlas. We used the radioligand [^18^F] FPEB in the human cohort and [^3^H] MPEP in the animal study. [^18^F] FPEB is developed from the [^3^H] MPEP scaffold and binds the same receptor site as MPEP in a fully competitive manner [108]. [^3^H] MPEP can act as a positive allosteric modulator of mGluR4 as a mass dose of the unlabelled compound and this has only been shown in one study and only at high micromolar concentrations, which we did not use (> 10 mM)[109].

In conclusion, we investigated the excitatory/inhibitory balance in adults with ASD and specifically examined both receptor and neurotransmitter differences and the correlation between them. We have extended the previous pilot PET study and prior post-mortem work on mGluR5 [27, 65, 110] to show higher availability of total mGluR5 in humans with ASD and we recapitulated these findings in *Cntnap2* KO mice, a mouse model associated with ASD. We also found that mGluR5 levels in the left striatal/thalamic ROI were tightly linked to GABA + levels in the same location. Evidence from animal models points towards developmental deficits in mGluR5 impacting upon GABAergic cells and interneurons. Our preliminary results suggest the possibility that mGluR5 increases as a (VPFX method (3D time of flight iterative reconstruction): This is also referred to as OSEM (Ordered subset expectation maximization), the most often used PET image reconstruction algorithm, which is an iterative statistical algorithm [111]. Because image reconstruction is an ill-conditioned problem, image noise increases with number of iterations. To mitigate image noise, the OSEM algorithm is usually stopped before it has converged; additionally, the images are often post-smoothed using various filters [112]. Iterative reconstruction is slow and results in non-uniform convergence and salt & pepper noise but provides high resolution.) compensatory response to deficits in GABAergic development. However, we cannot exclude the possibility that the higher availability of mGluR5 drives lower levels of GABA + in the left striatum/thalamus. The same finding of higher binding potential in both - two separate, albeit pilot - studies adds weight to the suggestion that this finding may be a key feature of ASD. Furthermore, based on our results we suggest using the *Cntnap2* mouse model to examine how modulating mGluR5 relates to GABA + and the behavioural phenotype to inform further human studies.

## Supplementary information

Supplementary material
