# Supplementary material for: From bench to bedside: The mGluR5 system in people with and without Autism Spectrum Disorder and animal model systems
Source: Transl Psychiatry. 2022 Sep 20;12:395. doi: 10.1038/s41398-022-02143-1 (PMC9489881; doi:10.1038/s41398-022-02143-1)
Supplement: Supplementary file 1 — Supplementary material [file 41398_2022_2143_MOESM1_ESM.pdf]

From bench to beside: The mGluR5 system in people with and without Autism Spectrum Disorder and animal model systems

Supplementary material

Cornelia Carey MRCPsych[1], Nisha Singh PhD[2,3], Joel T. Dunn PhD[4], Teresa Sementa MCh[3,4], Maria Andreina Mendez PhD[1], Hester Velthuis MSc[1,5,6], Andreia C. Pereira PhD[1,7], Charlotte Marie Pretzsch PhD[1], Jamie Horder PhD[1], Stefan Hader PhD[4], David J Lythgoe PhD[2], Diana-Georgina Rotaru MSc[2], Anthony Gee PhD[4], Diana Cash PhD[8], Mattia Veronese PhD[2], Declan Murphy MD[1,5,9]*, Grainne McAlonan PhD[1,5,6]*

1. Department of Forensic and Neurodevelopmental Sciences, Institute of Psychiatry, Psychology and Neuroscience, King's College London, De Crespigny Park, London SE5 8AF, United Kingdom.
2. Department of Neuroimaging, Institute of Psychiatry, Psychology & Neuroscience, King's College London, De Crespigny Park, London SE 5 8AF, United Kingdom.
3. School of Biomedical Engineering & Imaging Sciences, 4th floor Lambeth Wing, St Thomas' Hospital, King's College London, London SE1 7EH, UK.
4. PET Centre, School of Biomedical Engineering and Imaging Sciences, St Thomas' Hospital, King's College London SE1 7EH, United Kingdom.
5. NIHR Maudsley Biomedical Research Centre, Institute of Psychiatry, Psychology & Neuroscience, King's College London, De Crespigny Park, London SE 5 8AF, United Kingdom.

6. Autism Assessment and Behavioural Genetics Clinic, South London and Maudsley NHS Foundation Trust, Bethlem Royal Hospital, Beckenham, UK.

7. Coimbra Institute for Biomedical Imaging and Translational Research (CIBIT), ICNAS, Polo 3 Azinhaga de Santa Comba 3000-548 Coimbra, Portugal.

8. Centre for Biomarker Research and Imaging for Neuroscience, James Black Centre, Denmark Hill, London, United Kingdom.

9. Sackler Institute for Translational Neurodevelopment, Institute of Psychiatry, Psychology and Neuroscience, King's College London, London, UK.

*Senior authors

Corresponding author: Cornelia Carey, Department of Forensic and Neurodevelopmental Sciences, Institute of Psychiatry, Psychology and Neuroscience, King's College London, De Crespigny Park, London SE5 8AF, United Kingdom. Email: corneliacarey1@gmail.com; Phone: +353 872906927

PET participant demographics and clinical scores.

Table 1: Values are shown as Mean ± Standard Deviation. P-values are the outcome of independent sample t-tests testing for a difference in group means.

Measure	Control	ASD	P-value (t-test)
**Number**	9	6	NA
**Age**	34.7 +/- 13.0	30.7 +/- 10.0	0.54
**Full Scale IQ**	122.9 +/- 12.4	118.2 +/- 13.8	0.56
**ADOS**	NA	7.7	NA
**AQ**	14.0 +/- 4.2	33.5 +/- 20.5	0.3
**OCI-R**	5.5 +/- 4.0	27.7 +/- 6.1	<0.0001*
**Barkley child**	12.6 +/- 7.5	37.7 +/- 24.9	0.2
**Barkley adult**	2.0 +/- 2.8	25.7 +/- 12.0	0.08
**BDI**	2.8 +/- 3.4	12.0 +/- 11.6	0.2
**SSAI pre-PET scan**	26.3 +/- 5.1	39.2 +/- 14.8	0.1
**BFRS**	4.5 +/- 5.2	19.5 +/- 13.4	0.1
**AUDIT**	3.25 +/- 1.75	4.2 +/- 2.8	0.46

Medication profile for PET participants.

Table 2: Nine participants were medication free. Three autistic participants and three controls admitted to the use of routine medication.

Participant	ASD vs controls	Medications
1	ASD	Nil
2	ASD	Nil
3	ASD	Nil
4	ASD	Salbutamol
5	ASD	Ibuprofen, Zopiclone, Propanolol
6	ASD	Antihistamines
7	Control	Omeprazole
8	Control	Antihistamine
9	Control	Antihistamine
10	Control	Nil
11	Control	Nil
12	Control	Nil
13	Control	Nil
14	Control	Nil
15	Control	Nil

PET Data Acquisition & Processing Methods

Trace & Patient Preparation

The radioligand [^{18}F] FPEB was synthesised as previously described and a dose of 200 MBq was injected in the participants' dominant antecubital vein as a 10 second bolus. Prior to image acquisition subjects had an arterial cannula inserted in the opposite wrist to the tracer cannula for arterial blood sampling. Subjects were positioned supine on the PET-CT scanner (GE Discovery 710) with their head in a moulded head rest to minimize movement.

Scan Acquisition

A low-dose computed tomography was acquired immediately prior to the PET scan for attenuation correction and PET emission data was collected in 3D and list-mode for a duration of 90 minutes, starting 10 seconds prior to tracer injection.

Blood Sampling

Continuous and manual arterial blood samples were also acquired during the scan for deriving whole blood and plasma-parent concentrations for input functions to the kinetic modelling. Continuous blood samples were acquired for the first 15 minutes following PET scan start with the arterial line (PTFE coated, inner diameter 1mm) connected to an automated blood sampling system (Allogg ABSS, www.allogg.se, Sweden) and a peristaltic pump set at 5mL/min. Manual samples were acquired from a three-way tap close to the Allogg detector. Seven 6mL samples were drawn at 3, 8, 12, 20, 30, 60- and 90-minutes post scan start. These samples were used to measure whole blood tracer concentrations and centrifuged for plasma concentrations to be measured and metabolite-parent separation. Five additional 0.5mL manual samples were acquired at 5, 10, 14, 45 and 75 minutes to improve Allogg-PET cross-calibration and extrapolating the whole-blood continuous curve to 90 minutes.

Blood Metabolite Processing

Arterial blood samples were collected in dipotassium EDTA (ethylenediaminetetraacetic) lined blood tubes (BD #367873) at specific time intervals as listed above. 200 µL of blood was removed for gamma counting. The remainder was centrifuged at ~ 3000 g at room temperature for 5 minutes to separate the plasma. To separate and quantify the parent radiotracer from the metabolites, the remaining plasma (~4 mL) was treated with 8 M urea and 50 mM citric acid and loaded on to the capture column (19 x4.6 mm^2, Phenomenex SPE C18 Strata-X sorbent) on a high-performance liquid chromatography (HPLC) system (Agilent 1200 series) similar to Hilton and colleagues' Column-Switching HPLC method for the Analysis of Plasma in PET Imaging Studies, Nuclear Medicine & Biology (1). An isocratic gradient of 1% *v/v* acetonitrile in H_2O at a flow rate of 4 mL/min was applied and, 2 minutes later, the trap activity on the capture column was back-flushed onto an analytical column (Luna® 5 µm C18(2) 100 Å, 250 × 4.6 mm column; Phenomenex# 00G-4252-E0). Elution was carried out using an isocratic gradient of 60% *v/v* acetonitrile in 50 mM ammonium acetate (pH 6.8) at a flow rate of 1.25 mL/min and 1 mL fractions were collected for the duration of the run. HPLC detection was carried out using a serial UV detector (λ = 254 nm) and the collected fractions were counted on a calibrated gamma-counter (Wizard2 2470, Perkin-Elmer). Continuous blood data was calibrated using overlapping manual whole-blood samples.

Image Processing

PET images were reconstructed on the PET-CT scanner using the VPFX method (fully 3D time-of-flight iterative reconstruction) with frame durations: 1 x 10 seconds, 10 x 5 seconds, 6 x 10 seconds, 3 x 20 seconds, 87 x 60 seconds. The VPFX method is also referred to as OSEM (Ordered subset expectation maximization), the most often used PET image reconstruction algorithm, which is an iterative statistical algorithm (2). Because image reconstruction is an ill-conditioned problem, image noise increases with number of iterations. To mitigate image noise, the OSEM algorithm is usually stopped before it has converged; additionally, the images are often post-smoothed using various filters (3). Iterative reconstruction is slow and results in non-uniform convergence and salt & pepper noise but provides high resolution. Dynamic images were reconstructed with attenuation correction (AC) and without (NAC). AC-PET images were also corrected for scatter and geometry corrections. All dynamic data was decay corrected within and across frames.

Frame-by-frame motion correction was applied by using the rigid-body transforms found using the 'realign' function in SPM12 (https://www.fil.ion.ucl.ac.uk/spm/) applied to the NAC frames

(ignoring the first 50 seconds of data to avoid low counts). These transforms were then applied to the AC PET images. Structural MRI images (MPRAGE) for each subject were segmented using the 'unified segmentation' algorithm in SPM12. These outputs were co-registered to a summed AC PET image (3-42 minutes) using SPM12 to provide the probabilistic grey matter masks for each subject in PET image space. The CIC atlas was warped and co-registered to the subject's PET image using the warping and co-registration parameters calculated from the structural MRI.

Mean time activity curves (TAC) weighted for grey matter were extracted from the motion corrected dynamic AC PET images for whole brain, left striatum/thalamus & dorsomedial prefrontal cortex. A mask of the MRS voxel-of-interest was used to define the ROIs (left striatum/ thalamus). The TAC from the cerebellum region identified from the co-registered CIC atlas was also extracted.

Blood Data Processing

All blood, plasma and metabolite samples were measured in a Perkin-Elmer Wizard2 2470 10 detector gamma-ray counter (https://www.perkinelmer.com/) cross calibrated with the PET scanner. All counted data was measure with a fixed-energy window and corrected for decay, background, and detector crosstalk. Blood and plasma samples were measured as 0.2mL volumes and metabolite fraction in volumes under 1mL.

Prior to further processing, all blood data was delay corrected to the start of the whole brain TAC rise (measured visually). Continuous blood data was cross calibrated using nested whole blood manual sample measurements. Continuous blood data was extrapolated between 15-90 minutes using a constrained decaying multiple exponential function using a non-negative least-square approach fitted to the manual blood sample data. This data was used as the whole-blood input function.

Plasma concentrations were calculated by multiplying the whole-blood input function by the mean plasma-over-blood ratio calculated from each subject's manual samples.

Plasma parent fractions (ratio of FPEB to all 18F labelled activity) were fitted to the sigmoid function described by Owen et al (2014) (4). The Plasma Parent concentration was calculated by multiplying this fitted parent fraction model with the plasma concentration. This data was used as the plasma-parent input function.

Kinetic modelling

Volume of Distribution (VT) and Distribution Volume Ratio (DVR) were calculated using the multilinear regression models used previously (5). VT was calculated using the "MA1" method described by Ichise et al (6). DVR was calculated using the "MRTM1" method described by Ichise el al (7) using the cerebellum TAC as the reference region. Both methods started the fits at t = 30 min. Data for the MRTM1 method was cropped at 76 minutes to allow for the patient with the short scan to be included.

Software

All data processing and model fitting was performed using Matlab 2017a (www.mathworks.com) with in-house code unless otherwise stated.

Individual Positron Emission Tomography Time Activity Curves & Model Fits.

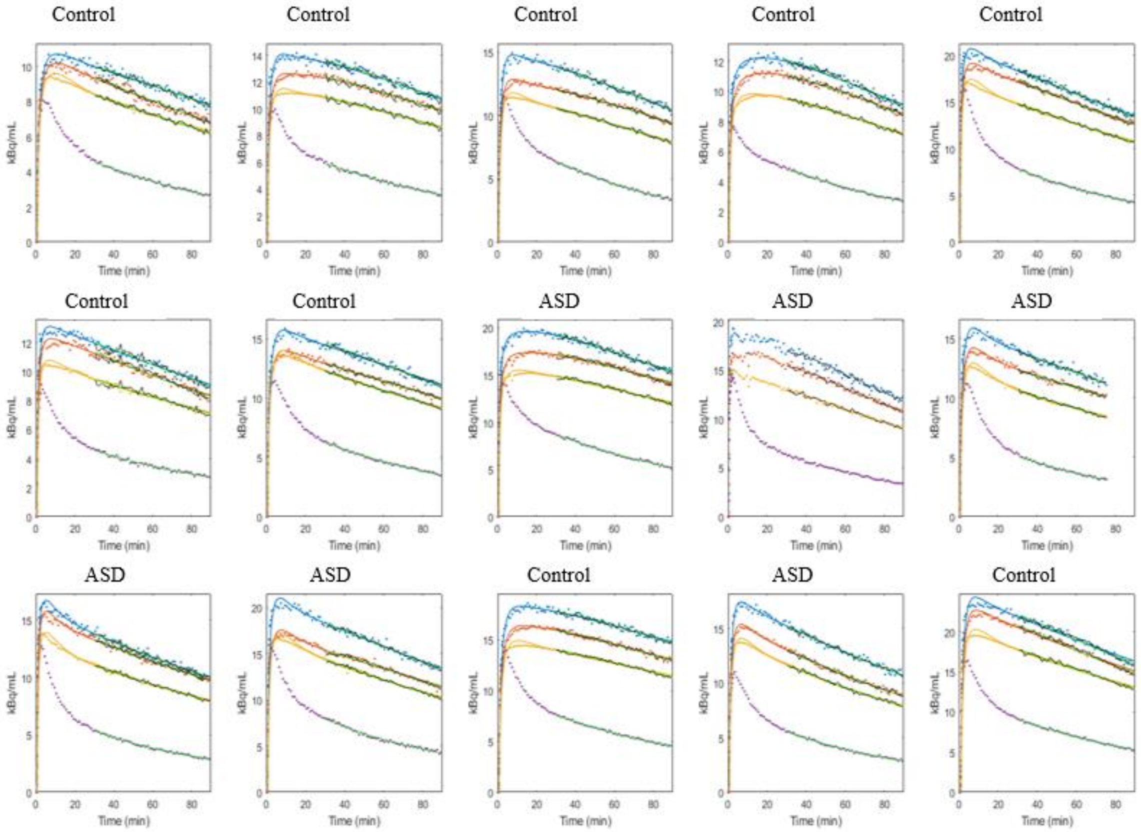

Figure 1: Dots = ROI TACs: blue = left striatum, red = DMPFC, yellow = whole brain, purple = cerebellum. Matched coloured solid line = 2TCM-CBL fit. Green solid line = MA1. Black solid line = MRTM1.

Dorsomedial prefrontal cortex

Human study

The two groups did not differ significantly for age and IQ. As expected, there were significantly higher obsessive-compulsive behaviours as rated on the Obsessive-Compulsive Inventory – Revised (OCI-R) in the ASD group compared to the non-ASD group.

^{1}H-MRS study

There was no difference in voxel tissue composition for grey matter($t(13)=1.48$, $p=0.16$), white matter ($t(13)=1.28$ $p=0.22$), or CSF ($t(13)=0.80$, $p=0.44$) between the two groups. There were no significant differences between groups for %CRLB for GABA ($t(13)=0.13$, $p=0.90$) or %CRLB for Glx ($t(13)=0.34$, $p=0.74$). There was no evidence of group differences in estimated Glx ($t\ (12) =0.63$, $p=0.54$) or GABA+ ($t\ (13) =0.52$, $p=0.61$) levels in the dorsomedial prefrontal cortex. See Figure 2.

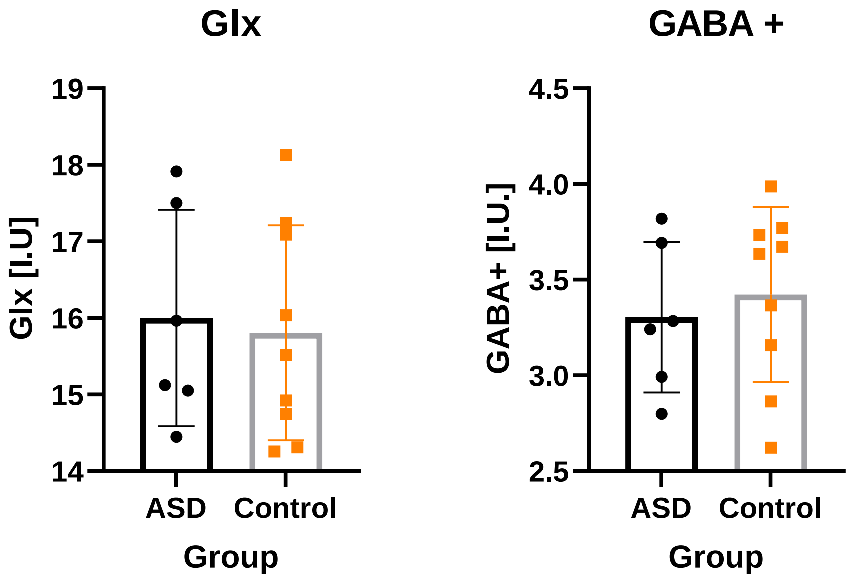

Figure 2: DMPFC Glx and GABA+ complex between groups. Error bars represent one standard deviation.

[^{18}F] FPEB: No group difference in mGluR5 receptor availability

Our analysis showed no difference in mGluR5 availability as measured using DVR in specific [^{18}F] FPEB binding in the MRI delineated dorsomedial prefrontal cortex mask ($t(13)=1.34$, $p=0.20$). See Figure 3.

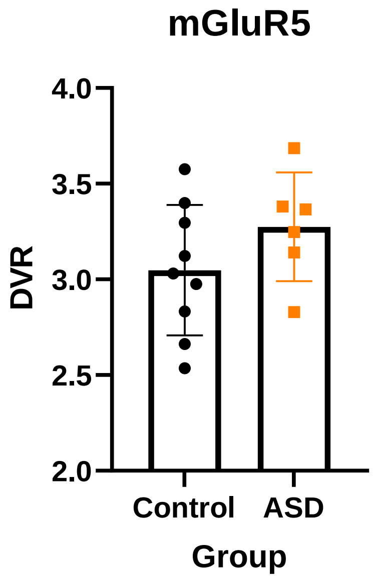

Figure 3. Frontal mGluR5 binding potential between groups. Error bars represent one standard deviation.

Quantitative autoradiography findings

There was no significant difference between the ASD mouse models and wild type mice for any of the three strains: Cntnap2, Shank3 deletion and 16p11.2 deletion.

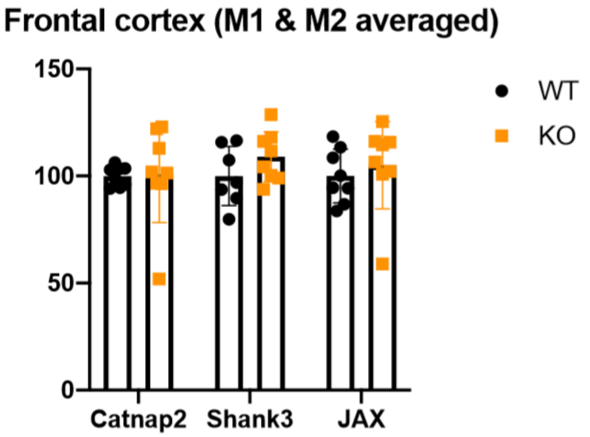

Source of Variation	% of total variation	P value	P value summary	Significant?
Interaction	1.272	0.7603	ns	No

	Significant?	P value
Catnap2	No	0.918962
Shank3	No	0.264732
JAX	No	0.515740

Figure 4: Animal frontal cortex: All three strains

References

1. Hilton J, Yokoi F, Dannals RF, Ravert HT, Szabo Z, Wong DF. Column-switching HPLC for the analysis of plasma in PET imaging studies. Nucl Med Biol. 2000;27(6):627-30.
2. Lantos J, Mittra ES, Levin CS, Iagaru A. Standard OSEM vs. regularized PET image reconstruction: qualitative and quantitative comparison using phantom data and various clinical radiopharmaceuticals. Am. J. Nucl. Med. Mol. Imaging. 2018;8(2):110-8.
3. Tong S, Alessio AM, Kinahan PE. Image reconstruction for PET/CT scanners: past achievements and future challenges. Imaging Med. 2010;2(5):529-45.
4. Owen DR, Guo Q, Kalk NJ, Colasanti A, Kalogiannopoulou D, Dimber R, et al. Determination of [(11)C]PBR28 binding potential in vivo: a first human TSPO blocking study. J Cereb Blood Flow Metab. 2014;34(6):989-94.

5. Sullivan JM, Lim K, Labaree D, Lin SF, McCarthy TJ, Seibyl JP, et al. Kinetic analysis of the metabotropic glutamate subtype 5 tracer [(18)F]FPEB in bolus and bolus-plus-constant-infusion studies in humans. J Cereb Blood Flow Metab. 2013;33(4):532-41.
6. Ichise M, Toyama H, Innis RB, Carson RE. Strategies to improve neuroreceptor parameter estimation by linear regression analysis. J Cereb Blood Flow Metab. 2002;22(10):1271-81.
7. Ichise M, Liow JS, Lu JQ, Takano A, Model K, Toyama H, et al. Linearized reference tissue parametric imaging methods: application to [11C]DASB positron emission tomography studies of the serotonin transporter in human brain. J Cereb Blood Flow Metab. 2003;23(9):1096-112.
